# Association Between Long-Term Proton Pump Inhibitor Therapy and Vitamin B12 Status: A Systematic Review and Meta-Analysis

**DOI:** 10.7759/cureus.90038

**Published:** 2025-08-13

**Authors:** Oliver Parnham, Wesley Patient

**Affiliations:** 1 Medicine, University of Warwick, Coventry, GBR; 2 Gastroenterology, University Hospital Coventry and Warwickshire NHS Trust, Coventry, GBR; 3 Anaesthesiology, Royal Devon University Healthcare NHS Trust, Exeter, GBR

**Keywords:** homocysteine, ppi, proton pump inhibitor, vitamin b12, vitamin b12 deficiency

## Abstract

Proton pump inhibitors (PPIs) are widely prescribed medications that have been linked to vitamin B12 deficiency. However, due to methodological differences between studies and the diagnostic inaccuracies of vitamin B12 assessment methods used, results are conflicting. This systematic review and meta-analysis investigated the association between chronic (>6 months) PPI use and vitamin B12 deficiency by assessing two biomarkers of vitamin B12 status: serum vitamin B12 and total homocysteine levels. A systematic search was conducted using PubMed, Cochrane Library, and Google Scholar. Data was extracted from each study and used to calculate standardised mean differences and 95% confidence intervals (95% CI) for serum vitamin B12 and total homocysteine levels between chronic PPI users and controls using a random effects model. Heterogeneity was tested via chi-squared, with p<0.05 indicating significant heterogeneity, and the degree of heterogeneity was quantified using the I² statistic. Six studies (1587 cases and 2272 controls), consisting of three cross-sectional studies, two cohort studies, and one randomised controlled trial, met the inclusion criteria. The standardised mean differences for serum total vitamin B12 (0.01, 95% CI -0.14-0.16, p=0.92) and total homocysteine levels (0.05, 95% CI -0.02-0.11, p=0.17) showed no difference between PPI users and controls. There was no association between chronic PPI use and vitamin B12 deficiency when assessing total serum vitamin B12 and total homocysteine levels. A lack of studies using multiple vitamin B12 biomarkers makes it difficult to recommend monitoring of vitamin B12 levels in low-risk patients prescribed chronic PPIs. Therefore, future studies should include at least two biomarkers of vitamin B12 status (serum vitamin B12, homocysteine, methylmalonic acid, and holotranscobalamin), in addition to serum folate, serum calcium, and dietary intake, across several age brackets and PPI dosages.

## Introduction and background

Proton pump inhibitors (PPIs) are widely prescribed, efficient, low-toxicity medications [1] used to treat acid-related diseases (e.g., dyspepsia, *Helicobacter pylori *infection, and gastroesophageal reflux) [2] and peptic ulcer prevention from non-steroidal anti-inflammatory drug (NSAID) use [3]. However, chronic PPI use has been linked with long-term adverse effects, including community-acquired pneumonia, hip fractures, and colorectal cancer [4] and, recently, with vitamin and mineral deficiencies, including Vitamin C, calcium, iron, magnesium, and vitamin B12 [5].

Vitamin B12 is an essential cofactor for DNA synthesis and cellular energy production and is almost exclusively found in animal-based and fortified foods [6]. In the stomach, it is absorbed after pepsin cleaves vitamin B12 from ingested food. Free B12 binds to haptocorrin (also known as transcobalamin-1 or R-protein) and moves into the duodenum, where pancreatic proteases cleave the B12-haptocorrin complex. Free B12 then binds to intrinsic factor, released in the stomach by parietal cells. The B12-intrinsic factor complex is absorbed by enterocytes in the terminal ileum [7] from where it is transported predominantly to the liver for long-term storage [8].

Prolonged vitamin B12 deficiency leads to megaloblastic anaemia and demyelinating neurologic disease, resulting in gait disorders and muscle weakness [9]. In addition, low vitamin B12 status has been linked to other aspects of neurological function, such as cognitive decline and visual disturbances [9]. However, subclinical B12 deficiency is more common and is linked to reductions in intake or insufficient absorption [10]. Evidence suggests that subclinical B12 deficiency is correlated with cognitive impairment, dementia, heart failure, and increased susceptibility to infection [11-14]. Martin et al showed that a time-limited window may exist for treating patients with cognitive dysfunction due to low serum cobalamin [15].

Proton pump inhibitors act by inhibiting the hydrogen/potassium ATPase enzymes on the parietal cells, preventing acid production. A low pH environment is required to activate pepsin, which is necessary to cleave vitamin B12 from ingested protein; thus, PPIs could impair B12 absorption via reduced gastric acid secretion [16]. PPIs may also cause bacterial overgrowth in the small intestine, leading to malabsorption and bacterial consumption of B12 [5]. It has also been previously proposed that PPIs may reduce intrinsic factor release; however, evidence has disproved this theory [17, 18].

Observational studies suggest that there may be an association between long-term PPI use and reduced circulating vitamin B12 levels [16], but a previous review emphasized that “evidence to date has been from small, poorly-controlled, nonrandomized retrospective studies or sporadic case reports with varying methods for measuring B12 levels and deficiency” [16]. A meta-analysis of five studies by Jung et al [19] showed an association between long-term acid-lowering drugs (H2 receptor antagonists and PPIs) and vitamin B12 deficiency, with a hazard ratio of 1.83, indicating a higher risk of B12 deficiency with chronic PPI use. The authors recommended monitoring of vitamin B12 levels in long-term PPI users. However, their criteria for what constituted vitamin B12 deficiency were not consistent between the different studies included. This disparity between vitamin B12 deficiency criteria is a limitation of the systematic review and meta-analysis by Jung et al [19], and despite their conclusion, conflicting views remain in the literature as to whether chronic PPI use increases the risk of vitamin B12 deficiency [20-28]. A more recent systematic review and meta-analysis was conducted by Choudhury and colleagues in 2023 [29], and as with Jung et al [19], they showed a potential link between vitamin B12 deficiency and PPI use, although they did state in their conclusion that the pooled odds ratio was too low to imply association, and therefore, further, better-designed studies in long-term PPI users are needed [29]. Moreover, the systematic review by Choudhury et al only assessed serum Vitamin B12 levels to determine deficiency [29].

Vitamin B12 status can be measured using four biomarkers. Two direct biomarkers: total serum vitamin B-12 and holotranscobalamin (holoTC). Total vitamin B12 measures the amount of B12 bound to transcobalamin and haptocorrin. Transcobalamin delivers B12 to all tissues of the body, whereas haptocorrin only delivers B12 to the liver. This store is mobilised to maintain serum B12 values. Therefore, a serum B12 value above a deficiency cut-off point does not necessarily indicate adequate B12 status. Thus, vitamin B12 deficiency can be present in the absence of low serum vitamin B12 levels [30]. However, a low value may represent a chronic abnormality or prolonged low intake [31]. By contrast, holotranscobalamin reflects only the B12 bound to transcobalamin, making it a more specific and sensitive parameter than total vitamin B12 [32]. Additionally, there are two functional biomarkers: homocysteine (Hcy) and methylmalonic acid (MMA), which become elevated during vitamin B12 deficiency. Each single biomarker has limitations, and there is general agreement that vitamin B12 deficiency should be diagnosed using a combination of two or more biomarkers [33, 34].

The UK National Institute for Health and Care Excellence (NICE) 2024 guidelines [35] recommend the measurement of either total B12 (serum cobalamin) or active B12 (serum holotranscobalamin) as the initial test for suspected vitamin B12 deficiency, unless the patient is pregnant, in which case use active B12 as the initial test, or if recreational nitrous oxide use is the suspected cause of deficiency, then use serum methylmalonic acid (MMA) as the initial test, or plasma homocysteine (this requires referral to secondary care). NICE also highlights that cobalamin levels are not easily correlated with clinical symptoms. Furthermore, clinically significant vitamin B12 deficiency may be present even with cobalamin levels in the normal range, especially in the elderly [35].

We are unaware of any systematic reviews that have utilised at least two biomarkers of vitamin B12 status. Therefore, we conducted a systematic review and meta-analysis of studies that used two or more biomarkers of vitamin B12 status to examine the inconsistencies surrounding the relationship between chronic PPI use and Vitamin B12 status.

## Review

Materials and methods

Search Strategy

The present systematic review was performed according to “PRISMA guidelines” (Preferred Reporting Items for Systematic Reviews and Meta-analyses).

**Figure 1:**
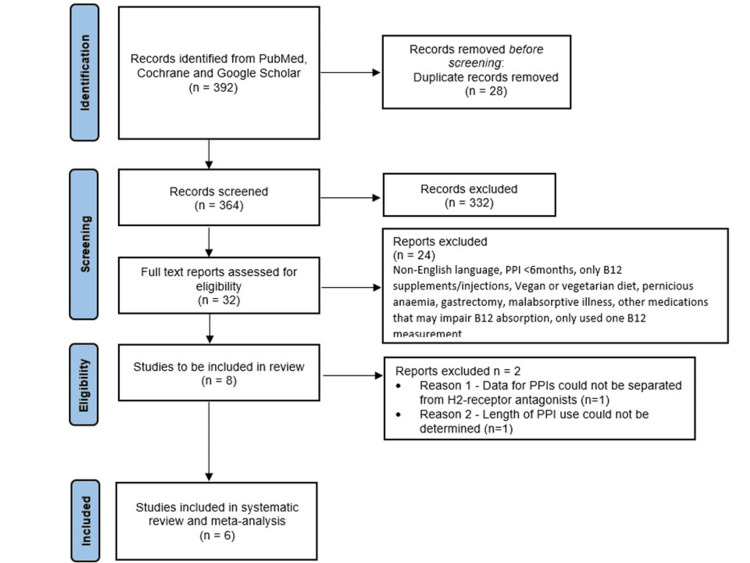
PRISMA flowchart PRISMA: Preferred Reporting Items for Systematic reviews and Meta-Analyses; PPI: proton pump inhibitor

A systematic review of the literature was conducted using PubMed, Cochrane Library, and Google Scholar to identify studies investigating PPI use and vitamin B12 status from January 2000 to October 2024. Search terms used were: ‘Proton Pump Inhibitors OR PPI OR Lansoprazole OR Omeprazole OR Esomeprazole AND Vitamin B12 OR Vitamin B12 deficiency OR B12 OR Cobalamin’. Searches were conducted independently by both authors. Each abstract was reviewed for relevance before the full article was read. Inclusion and exclusion criteria used to determine eligibility for studies included in this systematic review can be found in Table 1. Unpublished literature was not included. A PRISMA flowchart of the selection and screening method is provided in Figure 1. 

**Table 1:** Inclusion and exclusion criteria PPI: proton pump inhibitor

Inclusion Criteria	Exclusion Criteria
Observational studies (cohort, case-control and cross-sectional) or randomised controlled trials	Studies not written in English
Participants taking a regular PPI for at least 6 months	Patients taking a PPI for less than 6 months
At least two Vitamin B12 markers measured	Intake of any supplement containing Vitamin B12 (e.g., a multivitamin),
	Patients who receive vitamin B12 injections
	Consumption of a vegetarian or vegan diet after initial vitamin B12 measurements are taken prior to starting a PPI
	Pernicious anaemia
	Previous total or partial gastrectomy
	Congenital intrinsic factor deficiency (Imerslund Gräsback syndrome)
	Any intestinal condition causing malabsorption (e.g., gluten-induced enteropathy), ileal resection, Crohn’s disease, blind loop syndrome, parasites (e.g., giardiasis, fish tapeworm)
	Intake of any medication which may impair vitamin B12 absorption other than a PPI (i.e. Colchicine, H2-receptor antagonists, metformin) [35].

Data extraction

Data was extracted by both first authors independently, then reviewed for discrepancies to ensure accuracy, using a standardised data extraction form with Microsoft Excel (Microsoft Corporation, Redmond, USA). Data extracted included: 1) Study information (lead author, publication year, country of publication), 2) Study design (study type, study period, study setting, number of participants, number in intervention group, number of controls, inclusion and exclusion criteria, 3) Subject demographics (mean age, gender, average level of vitamin B12 biomarkers, average PPI use) 4) Study results, statistical significance, and study quality score. A summary of study parameters can be found in Table 2, which includes year of study, country conducted, study setting and population studied, study period, type of study, patient age range, and vitamin B12 biomarkers measured. A summary of data extracted that was used in the systematic review and meta-analysis can be found in Table 3, including the total number of participants, the number of PPI users (cases) and controls, the duration of PPI use, and the average serum vitamin B12 and total homocysteine values for each of the groups. 

**Table 2:** Study parameters included in the systematic review PPI: Proton pump inhibitor; MMA: Methylmalonic acid; HoloTC: Holotranscobalamin; NSAID: Non-steroidal anti-inflammatory.

Author	Date	Country	Study Setting	Study Period	Study Type	Patient Age Range	Biomarkers Measured
Den Elzen et al. [21]	2008	Netherlands	PPI users and their non-PPI using partners	3 years	Cross Sectional	≥65 y	Vitamin B12, Homocysteine
O’Leary et al. [22]	2011	Australia	Geriatric rehabilitation unit	1 year	Cross-sectional	≥60 years	Vitamin B12, MMA, Homocysteine
Porter et al. [23]	2021	Pan Ireland	Non-institutionalised adults	4 years	Cross-sectional	≥60 years	Vitamin B12, HoloTC, Homocysteine
Qorraj-Bytyqi et al. [25]	2018	Kosovo	Chronic NSAID users	1 year	Cohort	18-65 years	Vitamin B12, Homocysteine
Hirschowitz et al. [36]	2008	USA	Acid hyper secretors	18 years	Cohort	Unknown	Vitamin B12, MMA, Homocysteine
Galmiche et al. [37]	2011	11 European Countries	Gastro-oesophageal reflux sufferers	5 years	Randomised Trial	18-70 years	Vitamin B12, Homocysteine

**Table 3:** Data extraction of studies included in the systematic review Values are not present for the control group in the study by Hirschowitz et al. [36] as no control group was used in their study. PPI: Proton pump inhibitor; tHcy: total Homocysteine.

Author	Total Participants	PPI users	Controls	Duration of PPI Use	Average PPI B12 (pmol/L)	Average Control B12 (pmol/L)	Average PPI tHcy (µmol/L)	Average Control tHcy (µmol/L)
Den Elzen et al. [21]	250	125	125	≥3 years	345	339	12.6	13.1
O’Leary et al. [22]	49	27	22	≥2 years	295.8	232.7	15	14
Porter et al. [23]	2973	1207	1766	≥6 months	265.46	268	14.64	14.3
Qorraj-Bytyqi et al. [25]	209	167	42	≥1 year	393.5	416.2	14.5	13.8
Hirschowitz et al. [36]	41	41	0	>17 months	543.17	-	10.14	-
Galmiche et al. [37]	372	192	180	≥5 years	335.8	313	12.5	13.2

Statistical Analysis

Data was analysed using the Review Manager 5.4 software (The Cochrane Collaboration, London, UK). Pooled standardised mean difference and 95% confidence intervals (CI) were obtained using a random effects model. Heterogeneity was tested via chi-squared test, with P<0.05 indicating significant heterogeneity, and the degree of heterogeneity was quantified using the I² statistic, with values of 25%, 50% and 75% corresponding to low, moderate, and high degrees of heterogeneity, respectively. These analyses and statistical tests were repeated separately for each biomarker of vitamin B12 status. Publication bias was assessed by using funnel plots, Begg’s rank test, and/or Egger’s regression test, as appropriate.

Risk of Bias Assessment

Risk of bias was assessed using the Appraisal Tool for Cross-sectional studies (AXIS) (Institute of Health & Society, Newcastle University, Newcastle upon Tyne, UK) [38] for Cross-sectional studies, the Newcastle-Ottawa Scale (NOS) [39] for cohort studies, and the Cochrane Risk of Bias 2.0 tool (RoB 2.0; The Cochrane Collaboration, London, UK) [40] for randomised controlled trials. A summary of the results can be found in Table 4. The AXIS tool [38] assesses the risk of bias in six domains: study aims and objectives, study design, results, discussion, other (funding, conflicts of interest, and ethics), with an overall grading of low, moderate, or high risk of bias. The NOS [39] assesses the risk of bias in three domains: selection, comparability, and outcome, with an overall grading of good, fair, or poor quality. The RoB 2.0 tool [40] assesses risk across five domains: randomisation, deviations from intended interventions, missing outcome data, measurement of the outcome, and reported result, with an overall grading of low risk, some concern, or high risk. The papers were discussed and graded according to their respective risk of bias tool, with both authors needing to agree (OP and WP); discrepancies were resolved by author consensus. Using the appropriate tool for each respective study included in this review allowed the authors to undertake a comprehensive assessment of the risk of bias in the included studies, thus informing the overall quality of evidence presented in this review. 

**Table 4:** Risk of bias summary table RCT: Randomised Controlled Trial, RoB 2.0: Cochrane Risk of Bias 2.0 tool, NOS: Newcastle-Ottawa Scale, AXIS: Appraisal Tool for Cross-Sectional Studies.

Study ID	Study Design	Tool Used	Overall Risk of Bias
Den Elzen et al [21]	Cross-sectional	AXIS	Moderate risk
O’Leary et al [22]	Cross-sectional	AXIS	Low risk
Porter et al [23]	Cross-sectional	AXIS	Low risk
Qorraj-Bytyqi et al [25]	Cohort	NOS	Good quality (8/9)
Hirschowitz et al [36]	Cohort	NOS	Fair quality (6/9)
Galmiche et al [37]	RCT	RoB 2.0	Some concern

Results

Description of Selected Studies

The initial literature search found 363 studies after duplicate study removal. Abstracts were screened, resulting in 32 studies. On assessing full articles, eight studies met the eligibility criteria. On further analysis, the study by Valuck and Ruscin [41] was removed as it was not possible to separate Vitamin B12 markers from PPI and H2-receptor antagonist users. The cross-sectional study by Lerman et al was also removed from the final analysis due to no data indicating the length of PPI for participants included (the lead author was contacted for this information, but unfortunately, no response was received) [42], leaving six studies for the final analysis (Figure 1).

Of the remaining six studies, three were cross-sectional studies, two were cohort studies, and one was a randomised trial. A total of 4413 participants were included, with 1853 in the PPI group and 2560 in the control group. The mean age of participants was 64.6 years (46-80.4 mean years), and the ratio of male to female patients was almost equal at 47.1% male and 52.9% female on average across studies. All studies measured serum vitamin B12 levels and total homocysteine (tHcy). One study also measured holoTC, and a further two studies measured plasma MMA. As all studies measured serum vitamin B12 and tHcy, it was decided that it would be these biomarkers that would be compared in the final analysis. As no included study used standardised definitions for vitamin B12 deficiency, mean serum Vitamin B12 and tHcy were compared between cases and controls rather than determining rates of vitamin B12 deficiency. Furthermore, as only two studies, Qorraj-Bytyqi et al [25] and Porter et al [23], included baseline values of vitamin B12 markers prior to and following 6 months of PPI use, only post-PPI intervention values were used in the meta-analysis.

The study by Hirschowitz et al [36] had no control group; thus, their study data was not used in the meta-analysis. Where subgroups were present within PPI users for the studies by Hirschowitz et al [36] and Porter et al [23], the weighted means and pooled standard deviations were calculated for serum vitamin B12 and plasma Homocysteine levels. For Galmiche et al [37], only the 5-year B12 values were used, as a longer period taking PPIs should theoretically lead to a greater risk of vitamin B12 deficiency. For Porter et al [23], the 95% standard deviations were calculated as only the confidence intervals were presented in the data. No confidence intervals or standard deviations were available for Galmiche et al [37]; thus, their data were not used in the meta-analysis.

Results of Serum Vitamin B12 and Total Homocysteine Levels on PPI Users vs Controls

**Figure 2:**
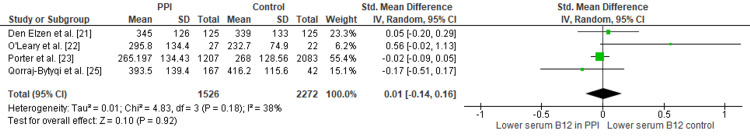
Forrest plot of standardised mean difference for serum vitamin B12 between PPI users and controls The forest plot above shows no significant difference in serum vitamin B12 levels between PPI users and controls (SMD 0.01, 95% CI: -0.04, 0.16, p: 0.92, I2: 38%). PPI: Proton pump inhibitor, SMD: Standardised mean difference, CI: confidence interval

**Figure 3:**
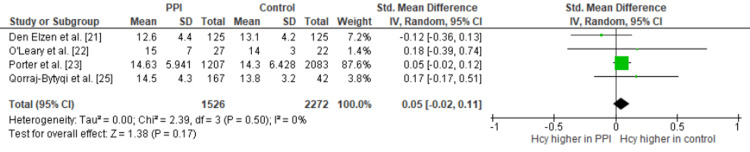
Forrest plot of standardised mean differences for homocysteine between PPI users and controls The forest plot above shows no significant difference in homocysteine levels between PPI users and controls (SMD 0.05, 95% CI: -0.02, 0.11, p: 0.17, I2: 0%). PPI: Proton pump inhibitor, Hcy: Homocysteine, SMD: Standardised mean difference, CI: confidence interval

A random-effects meta-analysis of four studies found no significant difference in serum vitamin B12 levels between PPI users and controls (Figure 2). The pooled standardized mean difference (SMD) was 0.01 (95% CI: -0.14 to 0.16, p = 0.92), indicating no meaningful effect in either direction (Figure 2). Heterogeneity was low to moderate (I² = 38%, p = 0.18), indicating some variability across studies, but not enough to significantly affect the overall estimate. Similarly, for total homocysteine (tHcy), no significant difference was observed between PPI users and controls. The pooled SMD was 0.05 (95% CI: -0.02 to 0.11, p = 0.17), favoring the control group but without statistical significance (Figure 3). There was no evidence of heterogeneity among studies (I² = 0%, p = 0.50). Both analyses were heavily weighted by the study by Porter et al. [23], which contributed the largest sample size and statistical weight to the pooled estimates.

Publication Bias

**Figure 4:**
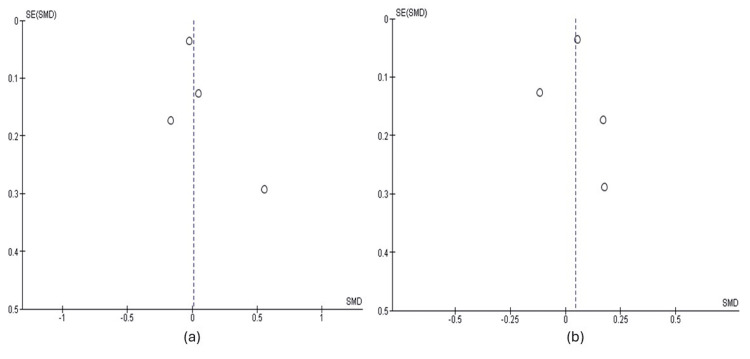
Funnel plots of the included studies (a) Funnel plot of studies included for serum B12; (b) Funnel plot of studies included for homocysteine. SE: standard error; SMD: standardised mean difference

Publication bias was not assessed as there were inadequate numbers of included trials to properly assess a funnel plot or to conduct advanced regression-based assessments, which are known limitations of the analytical methods of publication bias assessment [43]. However, based solely on the funnel plots provided (Figure 4), it appears no publication bias was present.

Discussion

This systematic review and meta-analysis aimed to explore the correlation between chronic PPI therapy and vitamin B12 status. Six eligible studies were included, with no statistically significant effect noted with at least 6 months of PPI medication on serum vitamin B12 or total homocysteine levels.

These findings differ from the meta-analysis by Jung et al [19], who found that use of PPIs for over 10 months had 1.83 times greater risk of vitamin B12 deficiency than non-PPI users. The contrasting results may be explained, in part, by differences in methodology. Our study measured differences in average serum B12 levels between PPI and non-PPI users, whereas Jung et al [19] measured the total number of B12-deficient patients. Furthermore, each study included in the meta-analysis by Jung et al [19] defined B12 deficiency differently. For example, the studies by Mitchell and Rockwood [44] and Force et al [45] did not define B12 deficiency, instead defining the parameter as any patient receiving Vitamin B12 replacement therapy. Lack of consistency in deficiency definitions is likely because “[d]efinitive cut-off points to define clinical and subclinical deficiency states are not possible, given the variety of methodologies used and technical issues” [30]. Therefore, we compared average serum B12 and total homocysteine levels between PPI users and a control group. This is particularly relevant as hyperhomocysteinemia can be present in the absence of low serum B12 levels, yet is associated with an increased risk of cardiovascular and thromboembolic disease [46, 47]. 

Jung et al [19] also recommended monitoring Vitamin B12 levels every 2-3 years in chronic PPI users. Whereas recent best practice advice from the American Gastroenterological Association does not recommend routine screening or monitoring of Vitamin B12 [48], and our analysis supports this guidance. Sheen and Triadafilopoulos [16] showed that a correlation existed between PPI use and lower serum Vitamin B12 levels, but this was only clinically relevant in elderly and malnourished patients. Therefore, routine screening may only be required in these higher-risk groups. This is further supported by the increased prevalence of atrophic gastritis in the elderly, which is often undiagnosed and has a statistically significant association with lower serum B12 [23]. Additionally, high rates of malnourishment are prevalent in the elderly [49]. Sheen and Triadafilopoulos [16] state that the lack of strong correlation between PPIs and vitamin B12 deficiency is likely due to there being no impact on intrinsic factor secretion, implying there is minimal effect on absorption of enterohepatically-recycled cobalamin or unbound cobalamin.

Studies used within this meta-analysis have reached differing conclusions. Porter et al [23] investigated the association between atrophic gastritis and PPI use with B12 status. They found serum B12 and homocysteine levels were not statistically different at any dose between PPI users and controls. Only at higher PPI doses, ≥30 mg/d, were significantly lower holoTC levels and a higher prevalence of vitamin B12 deficiency via the combined indicator B12 (cB12) found (25% compared with 15% in controls) [23]. It has been argued that holoTC levels are one of the most sensitive biomarkers for diagnosing B12 deficiency [50]. Unfortunately, due to the lack of studies measuring holoTC, we were unable to include this biomarker in our analysis. Moreover, Porter and colleagues [23] found that regular consumption of fortified foods was associated with a lower risk of vitamin B12 deficiency, which presents a methodological barrier when assessing a correlation between PPIs and vitamin B12 in the absence of detailed information about dietary intake. 

The possible confounding variable of dietary intake may be somewhat assessed from the study by Den Elzen et al [21], who measured the vitamin B12 and homocysteine levels of 125 PPI users and their partners who acted as the control group, with all participants aged over 65 years. This study had the unique approach of using the participants' partners as the control group, minimising variation in dietary intake between the groups. They found no statistically significant differences between the experimental and control groups for serum B12 and homocysteine levels.

In the randomised controlled trial by Galmiche et al [37], serum B12 and homocysteine levels in PPI users were measured at baseline, 1-, 3-, and 5-years post-PPI commencement in patients with chronic symptomatic Gastro-oesophageal reflux disease (GORD) across eleven European countries. Vitamin B12 deficiency was not a primary outcome of this study; however, both serum B12 and homocysteine were measured as part of the safety assessment of PPI use versus laparoscopic anti-reflux surgery. Although Galmiche et al [37] do not provide standard deviations or confidence intervals for their B12 values, they state that no clinically relevant changes were noted. A compilation analysis of the study by Attwood et al [20] states that vitamin B12 and homocysteine levels remained constant, and abnormal values found were not published in the data as the authors deemed them not “clinically important”. Although it is difficult to interpret these results without standard deviations and confidence intervals, it appears there was no significant impact of PPIs on vitamin B12 levels in their study.

O’Leary et al studied 49 patients in a geriatric rehabilitation unit and found that there was no statistical difference between the 27 PPI users and 22 non-PPI users, and assessed participant dietary intake through dietary recall for the previous 12 months [22]. They found an interesting association between increased calcium and protein intake and a decreased risk of vitamin B12 deficiency with PPI use, which corresponds to findings observed in other studies [24, 51]. This presents potentially significant confounding variables that may not have been completely considered in the literature up to this point. O’Leary et al [22] indicate that the lack of effect on B12 markers in those taking PPIs was likely due to a higher intake of meats, eggs, and dairy foods.

Qorraj-Bytyqi et al [25] took 209 adults with osteoarthritis taking NSAIDs but no PPI, and assigned them to one of four different PPIs, to be taken for 12 months before assessing B12 markers. 42 matched adults were used as a comparison group. They found no statistically significant change in homocysteine, but there was a trend towards increased levels in the PPI groups, with increased homocysteine levels seen in 39.5% of PPI users compared to 19% of non-PPI users. A statistically significant reduction in serum vitamin B12 levels was seen for the PPI groups after 12 months (433.6 to 393.5 pg/ml), but only 3% of participants were classed as B12 deficient, defined as serum B12 less than 191 pg/ml by the authors. Inclusion of PPI dosages used for each of the subgroups is a strength of their study [25]; however, the dosages may have been too low to see considerable vitamin B12 changes within 12 months.

Hirschowitz et al [36] studied 61 patients with acid hypersecretion, 46 of whom had Zollinger-Ellison syndrome, treated with lansoprazole for between <3 and ≥12 years, using a median dose of 75 mg/day. They found that six patients had low serum B12, and 15 had elevated homocysteine levels with normal B12 and folate. However, the acid suppression caused by PPIs was not considered to be sufficient to affect B12 absorption [36]. It must be noted that this conclusion was made for hyper-secretors with a more acidic stomach environment at baseline and thus may not provide generalisable results to normo-secretors with GORD.

This current review has important limitations. Firstly, some studies included didn’t report B12 levels alongside demographic data, so we are unable to comment on the effect of Vitamin B12 levels in different age brackets. Equally, only one of the studies provided data for Vitamin B12 levels at different doses of PPIs, making it very difficult to explore the possibility of a dose-dependent relationship [23]. This review is heavily weighted by data from Porter et al [23], with over 60% of the data used coming from their study. A further limitation of this meta-analysis is the inclusion of only four studies, which restricts the statistical power and limits the ability to detect heterogeneity [52, 53], with observational studies increasing the risk of bias [54]. Furthermore, in several of the studies used, there was no data provided for the length of time a participant had been taking PPIs, only the minimum amount of time for all participants. However, this did allow us to ensure all participants were chronic PPI users (>6 months). Finally, raised homocysteine is not a marker specific to vitamin B12 status and is influenced by other nutrients, in particular folate deficiency and vitamin B6 deficiency, and other factors such as renal failure and hypothyroidism, and dietary factors such as alcohol intake [30, 55, 56]. Only two studies [23, 37] measured folate levels, but Galmiche et al [37] did not present the quantitative values due to data-compatibility issues, although a later review stated that levels were consistent with those of the SOPRAN study [20]. MMA is a more specific secondary marker than homocysteine [30] and has a greater correlation to cognitive decline in older adults [57]. Moreover, NICE recommend MMA in preference to homocysteine levels when assessing those that have worsening symptoms whilst receiving vitamin B12 replacement, likely due to the time critical requirements when testing plasma Hcy which needs to be put on ice and tested within 30-60 minutes of being taken, and the influence of folate deficiency on its value [35, 58]. However, MMA is not without its limitations, especially its reduced accuracy in the elderly and those with impaired renal function [34]. MMA is also a costly test, ranging from £11 to £80; however, increased frequency of MMA testing might be balanced out by fewer primary care appointments, reduced investigations, and reduced onward referrals, leading to earlier and more appropriate management of B12 deficiency [58]. The most appropriate first-line marker would be holoTC due to its higher positive and negative predictive value, with evidence supporting its higher accuracy in detecting vitamin B12 deficiency, particularly in the elderly, compared to serum cobalamin and MMA [32, 50, 56, 59]. Although indeterminate holoTC results may not preclude the need for additional confirmatory tests via MMA and Hcy [56, 60].

Utilising a combination of vitamin B12 markers by assessing serum cobalamin, holoTC, MMA, tHcy, and serum folate, in addition to serum calcium and dietary intake assessments (including alcohol intake), would be the optimal combination of markers for assessing vitamin B12 deficiency [55, 61, 62].

The present study found no statistically significant evidence that chronic PPI use affects vitamin B12 status and has highlighted the need for further research using adequate protocols, specifically multiple markers to quantify Vitamin B12 status with confidence.

## Conclusions

This systematic review and meta-analysis found that there was no association between chronic PPI use and vitamin B12 deficiency. We included studies that used average serum vitamin B12 levels and total homocysteine levels, which differs from previous systematic reviews. This systematic review is unable to comment on the effects across varying age groups, the effect of PPI over set time intervals, or the effect of varying doses of PPIs on vitamin B12 status. Large prospective studies are required to determine the effect of chronic PPI use on vitamin B12 and folate levels. The recommendation for future studies would be to measure at least two vitamin B12 biomarkers (serum vitamin B12, homocysteine, methylmalonic acid, and holotranscobalamin) in addition to serum folate, calcium, and dietary intake, regularly over a set period, across several age brackets and PPI dosages, and to use standardised criteria to quantify deficiency.
